# Base of the skull morphology and Class III malocclusion in patients with unilateral cleft lip and palate

**DOI:** 10.1590/2176-9451.20.1.079-084.oar

**Published:** 2015

**Authors:** Mariana Maciel Tinano, Milene Aparecida Torres Saar Martins, Cristiane Baccin Bendo, Ênio Mazzieiro

**Affiliations:** 1 PhD resident in Child and Adolescent Health, School of Medicine - Federal University of Minas Gerais (UFMG); 2 Postdoc resident in Pediatric Dentistry, Federal University of Minas Gerais (UFMG); 3 Assistant professor, Department of Orthodontics and Pediatric Dentistry, Federal University of Minas Gerais (UFMG); 4 PhD in Orthodontics, University of São Paulo (USP)

**Keywords:** Angle Class III malocclusion, Base of the skull, Cleft lip and palate

## Abstract

**OBJECTIVE:**

The aim of the present study was to determine the morphological differences in the base of the skull of individuals with cleft lip and palate and Class III malocclusion in comparison to control groups with Class I and Class III malocclusion.

**METHODS:**

A total of 89 individuals (males and females) aged between 5 and 27 years old (Class I, n = 32; Class III, n = 29; and Class III individuals with unilateral cleft lip and palate, n = 28) attending PUC-MG Dental Center and Cleft Lip/Palate Care Center of Baleia Hospital and PUC-MG (CENTRARE) were selected. Linear and angular measurements of the base of the skull, maxilla and mandible were performed and assessed by a single calibrated examiner by means of cephalometric radiographs. Statistical analysis involved ANCOVA and Bonferroni correction.

**RESULTS:**

No significant differences with regard to the base of the skull were found between the control group (Class I) and individuals with cleft lip and palate (P > 0.017). The cleft lip/palate group differed from the Class III group only with regard to CI.Sp.Ba (P = 0.015). Individuals with cleft lip and palate had a significantly shorter maxillary length (Co-A) in comparison to the control group (P < 0.001). No significant differences were found in the mandible (Co-Gn) of the control group and individuals with cleft lip and palate (P = 1.000).

**CONCLUSION:**

The present findings suggest that there are no significant differences in the base of the skull of individuals Class I or Class III and individuals with cleft lip and palate and Class III malocclusion.

## Abstract (pt)

**OBJETIVO:**

o objetivo do presente estudo foi determinar diferenças morfológicas da base do crânio de indivíduos portadores de fissura de lábio e palato e de má oclusão de Classe III, comparado-os com indivíduos controle com má oclusão de Classes I ou III.

**MÉTODOS:**

oitenta e nove indivíduos, de ambos os sexos, com idade variando entre 5 e 27 anos, Classe I (n = 32), Classe III não fissurados (n = 29) e Classe III com fissura labiopalatina unilateral (n = 28), oriundos do Centro de Odontologia e Pesquisa da PUC-MG e do Centro de Atendimento de Fissurados do Hospital da Baleia e da PUC-MG (CENTRARE), foram selecionados. Medições lineares e angulares da base do crânio, maxila e mandíbula foram realizadas e avaliadas por um único examinador calibrado, por meio de radiografias cefalométricas. Foram utilizados os testes ANCOVA e correção de Bonferroni para a análise estatística dos dados.

**RESULTADOS:**

com relação à base do crânio, os resultados não indicaram diferença estatística entre indivíduos controle (Classe I) e os indivíduos com fissuras (p > 0,017). O grupo com fissura foi diferente do grupo Classe III somente em relação à medida CI.Sp.Ba (p = 0,015). O comprimento maxilar (Co-A) apresentou diferença estatisticamente significativa na comparação entre o grupo controle (Classe I) e o grupo com fissuras (p < 0,001), sendo que os fissurados apresentaram uma maxila menor. Não foram encontradas diferenças na mandíbula (Co-Gn) entre indivíduos do grupo controle (Classe I) e indivíduos fissurados (p = 1,000).

**CONCLUSÃO:**

os resultados sugerem que não houve diferença estatisticamente significativa na base do crânio entre indivíduos Classe I e III e indivíduos com fissuras de lábio e palato com má oclusão de Classe III.

## INTRODUCTION

Correlations between the development of the base of the skull and maxillofacial components have been demonstrated in facial development studies.^1^^-^ 4 The morphology of the base of the skull may be an important factor in the anteroposterior relationship of the maxilla and mandible as well as in determining Class III malocclusion.^5^^,^ 6 ^,^ 7

Class III malocclusion results from a combination of morphological abnormalities of the base of the skull, maxilla and mandible as well as in vertical facial dimensions.^5^^,^ 8 ^-^ 11 Morphological variability in the craniofacial complex of individuals with Class III sagittal relationship suggests the influence of the base of the skull in the development of this type of malocclusion. Individuals with greater flexure of the base of the skull angle reveal a reduction in the horizontal dimension of the middle cranial fossa, with a consequent tendency toward nasomaxilllary retrognathism, a more forward positioning of the mandible and a prognathic craniofacial profile.^12^ Moreover, a lower angle between the ramus of the mandible and the base of the skull, a smaller and more retrognathic maxilla and a larger and more prominent mandible can lead to Class III malocclusion associated with Class III facial pattern.^11^^,^ 13

The development of the craniofacial complex in patients with cleft lip and palate has been studied in an attempt to establish the mechanisms and determinant factors of facial development in such individuals. A number of studies state that the base of the skull is intrinsically different in shape and size in patients with cleft lip and palate.^14^^-^ 18 This difference may affect the growth and positioning of facial structures, with an increased flexure of the base of the skull, thereby favoring the development of a Class III skeletal relationship. Nevertheless, other studies report that individuals with cleft lip and palate do not present significant differences in the base of the skull of which development is normal.^19^^,^ 20 ^,^ 21 Abnormalities in intermaxillary and interalveolar sagittal relationships in such patients may stem primarily from a reduction in the depth of the maxilla, with no changes in the rotation or length of the ramus of the mandible.^22^ Thus, the anteroposterior deformities often found in such individuals may actually result from surgical trauma, adaptive changes or a combination of both.

The literature does not reach a consensus regarding base of skull morphology in patients with unilateral cleft lip and palate. Additionally, there is considerable lack of current studies on this subject. For this reason, the aim of the present study was to compare the morphology of the base of the skull in individuals with unilateral cleft lip and palate and Class III malocclusion with control individuals with Class I and Class III malocclusion.

## MATERIAL AND METHODS

This study was approved by the Catholic University of Minas Gerais Institutional Review Board (PUC-MG) under protocol CAAE - 0012.0.213.000-07.

## Sample

The sample comprised 89 lateral cephalograms collected from the files of PUC-MG Dental Research Center and the Cleft Lip/Palate Care Center of Baleia Hospital and PUC-MG (CENTRARE). All cephalograms were taken from male and female patients at orthodontic treatment onset. Patients were aged between 5 and 27 years old (mean = 12.9; median = 12.0).

The sample was divided into three study groups: 1 - Control group comprising 32 cephalograms of Class I individuals with no history of orthodontic treatment; 2 - Group 2 comprising 29 cephalograms of Class III individuals with no history of orthodontic treatment; and 3 - Group 3 comprising 28 cephalograms of nonsyndromic, unilateral cleft lip/palate Class III individuals having undergone correction for cleft lip/palate at an early age (lip surgery at a mean age of 6 months, and palate surgery at a mean age of 18 months).

## Measurement methods

Cephalometric tracings were performed manually on acetate paper and based on patients' cephalograms. All tracings were performed by a single calibrated examiner. Intraexaminer agreement was assessed by paired Student's t-test. Linear and angular measurements were performed on two separate occasions with a 10-day interval in between. The p-value generated by the paired Student's t-test was 0.446 for linear measurements and 0.392 for angular measurements, thereby demonstrating no significant differences between measurements taken on the two different occasions.

**Figure 1 -:**
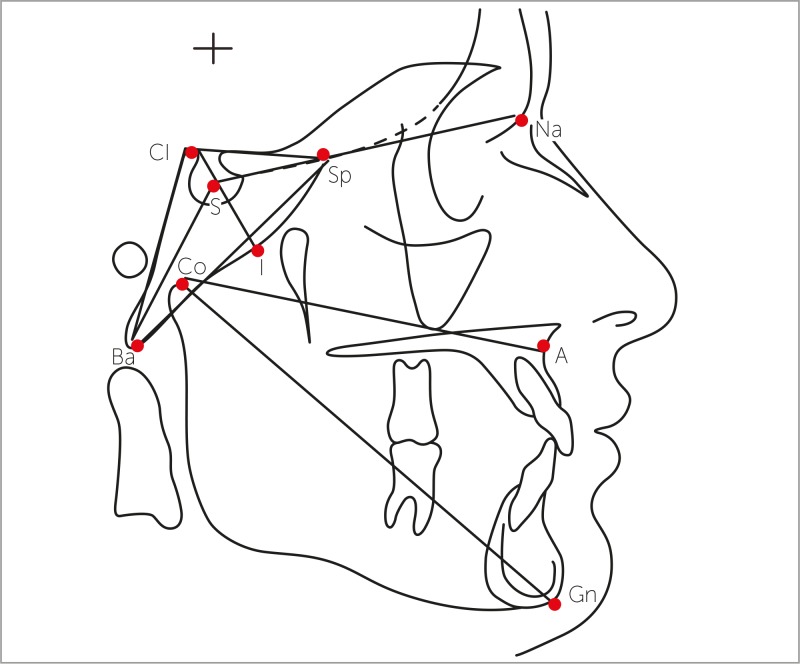
Cephalometric landmarks, linear and angular measurements.

The cephalometric landmarks used in the present study were as described by Jacobson:^23^ sella (S), nasion (N), basion (Ba), A-point (A), condyle (Co), gnathion (Gn), posterior clinoid process (Cl) and sphenoid (Sp). Linear (S-N, S-Ba, Co-A, Co-Gn, Ba-Cl, Sp-Cl, Ba-Sp and Cl-I) and angular (Ba.S.N, Ba.Cl.Sp, Cl.Ba.Sp and Cl.Sp.Ba) measurements were taken as shown inFigure 1. The height of the base of the skull (Cl-I) was measured by the distance of a straight line from Cl and S landmarks and a point intercepting the greater wing of the sphenoid bone at a point established as point I (I) (Fig 1).

## Data analysis

Statistical Package for Social Sciences (SPSS for Windows, version 19.0, SPSS Inc., Chicago, IL, USA) was used for data analysis. Initially, the three groups were analyzed with regard to age. As Shapiro-Wilk test determined that this variable was not normally distributed, Kruskal-Wallis test was used and revealed significant differences among the three groups with regard to age(P = 0.032).

Conversely, Shapiro-Wilk test determined that linear and angular measurements were normally distributed, for this reason, analysis of covariance (ANCOVA) was used for statistical analysis of data. Analysis of covariance is justified by the potential interference of age in the mean linear and angular measurements. In cases of significant differences among groups, Bonferroni correction was used to identify in which groups the difference was found. To prevent errors arising from multiple comparisons, the significance level (0.05) was divided by the number of comparisons;^24^ thus, p-values less than 0.017 were considered statistically significant (0.05 divided by 3).

## RESULTS

**Figure 2 -:**
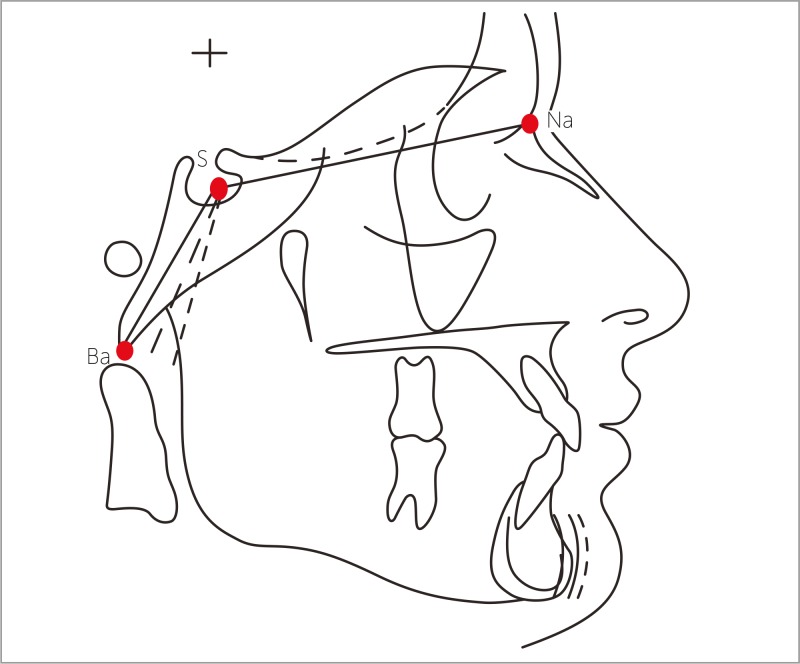
Ba.S.N angular measurements demonstrating flexure of the base of the skull angle.

Table 1 displays the angular measurements in the three groups. The cleft lip/palate group had intermediate measurements of the base of the skull that ranged between the control and Class III groups. No significant differences were found between the cleft lip/palate group and the control group (Class I). The cleft lip/palate group significantly differed from the Class III group only with regard to CI.Sp.Ba (P = 0.015). Significant differences in Ba.S.N, Ba.CI.Sp and CI.Sp.Ba were found between the control (Class I) and Class III groups. The lowest Ba.S.N was found in the Class III group, thereby indicating greater flexure of the base of the skull angle in comparison to the other groups (Fig 2).

**Table 1 -:** Mean angular measurements in different groups.

	Groups			P-value*			
Variable	Control (G1)	Class III (G2)	Cleft (G3)		Comparison between groups		
	Mean ± SD	Mean ± SD	Mean ± SD		G1 x G2	G1 x G3	G2 x G3
Ba.S.N	130.1 ± 5.0	125.6 ± 4.5	127.9 ± 5.0	0.002	0.001	0.275	0.192
Ba.Cl.Sp	114.7 ± 6.9	108.5 ± 6.9	113.2 ± 5.2	0.002	0.002	1.000	0.037
Cl.Ba.Sp	23.4 ± 2.6	25.2 ± 2.8	24.3 ± 2.7	0.080	-	-	-
Cl.Sp.Ba	42.1 ± 5.3	46.3 ± 5.5	42.5 ± 3.6	0.003	0.004	1.000	0.015

* Analysis of covariance adjusted for age. SD= standard deviation; G1= group 1; G2= group 2; G3= group 3. Bonferroni correction= P < 0.017; p-values in bold significant at 0.017.

Table 2 displays the linear measurements in the three groups. Mean Co-A (maxilla) was greater in the control group (Class I) (90.8 mm) and lower in the cleft lip/palate group (85.1 mm). This difference was statistically significant (P < 0.001). Considering a p-value lower than 0.017 as statistically significant (as determined by Bonferroni correction), no significant difference was found between the Class III group and the cleft lip/palate group (P = 0.032). Mean length of the mandible (Co-Gn) was greater in the cleft lip/palate group (116.3 mm) in comparison to the other groups. However, this difference was not statistically significant. While no significant differences were found with regard to angular measurements, the linear measurements of the base of the skull were lower, except for S-N and Ba-Sp.

**Table 2 -:** Mean linear measurements in different groups.

	Groups						
Variable	Control (G1)	Class III (G2)	Cleft (G3)	P-value*	Comparison among groups		
	Mean ± SD	Mean ± SD	Mean ± SD		G1 x G2	G1 x G3	G2 x G3
S-N	70.4 ± 5.1	68.5 ± 4.2	71.4 ± 4.9	0.591	_	_	_
S-Ba	46.5 ± 3.0	45.9 ± 3.0	45.3 ± 4.0	0.049	1.000	0.113	0.082
Co-A	90.8 ± 7.8	86.1 ± 6.0	85.1 ± 7.0	< 0.001	0.208	< 0.001	0.032
Co-Gn	114.7 ± 9.7	114.6 ± 10.9	116.3 ± 9.7	0.029	0.050	1.000	0.069
Ba-Cl	49.4 ± 3.6	49.2 ± 3.2	48.1 ± 4.5	0.051	-	-	-
Sp-Cl	29.3 ± 2.5	29.5 ± 2.7	29.1 ± 2.9	0.726	-	-	-
Ba-Sp	66.8 ± 3.5	64.3 ± 3.6	64.9 ± 5.5	0.072	-	-	-
Cl-I	25.1 ± 2.6	25.9 ± 2.4	24.6 ± 3.2	0.028	0.191	1.000	0.027

* Analysis of covariance adjusted for age. SD= standard deviation; G1= group 1; G2= group 2; G3= group 3. Bonferroni correction = P < 0.017; p-value in bold significant at 0.017.

## DISCUSSION

In the present study, no significant differences were found with regard to the linear measurements of the base of the skull (S-Ba, Ba-Cl, Sp-Cl, Ba-Sp and Cl-l) (P > 0.017), even though they were lower in the cleft lip/palate group in comparison to control (Class I). These results are in agreement with others studies^17^^,^ 20 ^,^ 25 reporting that shorter measurements may be attributed to the small body children with cleft lip/palate normally have.

No significant differences were found for S-N among groups; however, mean S-N was greater in the cleft lip/palate group in comparison to the other groups. This is in disagreement with others studies^15^^,^ 16 ^,^ 26 ^,^ 27 ^,^ 28 reporting lower S-N in children with cleft lip and palate, thereby suggesting a relative difference in the craniofacial morphology of such individuals. Nevertheless, the majority of the aforementioned studies included individuals with different types and degrees of cleft lip and palate, which may explain the divergent findings.

Significant difference was found in Co-A between the control (Class I) and the cleft lip/palate group (P < 0.001), as the former had the greatest whereas the latter had the shortest measurement among the three groups, thereby suggesting a deficiency in the effective length of the maxilla in this group. This result is in agreement with other studies^29^^-^ 33 reporting the effect of surgical procedures on the anteroposterior growth and development of the maxilla in children with cleft lip and palate due to the formation of fibrous scar tissue at the surgery site. However, it is not yet clear whether maxillary retrognathism may also be related to intrinsic development deficiencies in such individuals. In some studies,^25^^,^ 34 ^,^ 35 the maxilla of individuals with cleft lip and palate was reduced in size in both operated and non-operated groups, thus suggesting that maxillary retrognathism may not be related to surgical procedures only, but may also be due to intrinsic factors of the condition itself.

No statistically significant difference was found with regard to the linear measurement of mandibular length (Co-Gn) (P > 0.017), which is in agreement with other studies^21^^,^ 25 ^,^ 26 ^,^ 36 ^,^ 37 reporting that the mandible of individuals with cleft lip and palate is equal in length to that of individuals without this condition. Likewise, no significant differences were found between the control and the Class III malocclusion group, which is in disagreement with other studies^9^^,^ 10 concluding that mandibular length progressively increases with age of Class III individuals.

No significant difference was found in the base of the skull angle (Ba.S.N), particularly between control and cleft lip/palate group (P = 0.275). This is in agreement with previous studies^16^^,^ 19 ^,^ 20 ^,^ 25 ^,^ 27 reporting that the malocclusion found in this group is much more the result of maxillary retrognathism caused by surgical trauma than the presence of a more flexed base of the skull, thereby determining the emergence of mandibular prognathism. However, significant differences were found between the control and the Class III malocclusion group, with a smaller angle in the latter group (125.6^o)^. Other studies^8^^,^ 10 ^,^ 38 ^,^ 39 also report that Class III individuals have morphological abnormalities in the craniofacial complex, with a reduction in the angle formed by the anterior and posterior segments of the base of the skull. The posterior base of the skull (S-Ba) exerts significant influence in the emergence of mandibular prognathism. This mandibular rotation caused by reduction in the angle may indicate an increase in the length of the linear measurement Cl-I due to the base of the skull being represented by a triangle in this study. Thus, the greater height of the base of the skull in Class III individuals may be the consequence of greater flexure of this structure.

No significant differences were found between the control and the cleft lip/palate group regarding the angular measurements of the base of the skull (Ba.Cl.Sp, Cl.Ba.Sp and Cl.Sp.Ba), thereby confirming absence of morphological differences between the two groups. However, significant differences in Ba.Cl.Sp and Cl.Sp.Ba were found between the control and the Class III malocclusion group, thereby demonstrating morphological differences in the craniofacial complex of these two groups.

Based on the results of this study it is reasonable to assert that, the base of the skull in individuals with unilateral cleft lip and palate does not differ significantly from that of individuals with Class I malocclusion; its development is, therefore, normal. In contrast, craniofacial morphology in individuals with Class III malocclusion differs significantly from that of individuals with Class I malocclusion, thereby suggesting that structural alterations in this morphology may influence the emergence of Class III malocclusion.

## CONCLUSION

No significant differences in the base of the skull of Class I or Class III individuals and cleft lip/palate individuals with Class III malocclusion were found. Results suggest that Class III malocclusion in cleft lip/palate patients might be associated with the length of the maxilla, only.

## References

[1] Bjork A (1955). Base of the skull. Am J Orthod.

[2] Hopkins GB, Houston WJ, James GA (1968). The base of the skull as an aetiological factor in
malocclusion. Angle Orthod.

[3] Enlow D, Kuroda T, Lewis A (1971). The morphological and morphogenetic basis for
craniofacial form and pattern. Angle Orthod.

[4] Enlow D, McNamara JA (1973). The neurocranial basis for facial form and
pattern. Angle Orthod.

[5] Sanborn RT (1955). Differences between the facial skeletal patterns of
class III malocclusion and normal occlusion. Angle Orthod.

[6] Guyer EC, Ellis 3rd EE, McNamara JA, Behrents RG (1986). Components of class III malocclusion in juveniles and
adolescents. Angle Orthod.

[7] Chang HP, Liu PH, Tseng YC, Yang YH, Pan CY, Chou ST (2014). Morphometric analysis of the base of the skull in
Asians. Odontology.

[8] Singh GD, McNamara JA, Lozanoff S (1997). Finite element analysis of base of the skull in subjects
with class III malocclusion. Br J Orthod.

[9] Miyajima K, McNamara JA, Sana M, Murata S (1997). An estimation of craniofacial growth in untreated class
III female with anterior crossbite. Am J Orthod Dentofacial Orthop.

[10] Mouakeh M (2001). Cephalometric evaluation of craniofacial pattern of
Syrian children with Class III malocclusion. Am J Orthod Dentofacial Orthop.

[11] Chang HP, Hsieh SH, Tseng YC, Chou TM (2005). Cranial-base morphology in children with class III
malocclusion. Kaohsiung J Med Sci.

[12] Lavelle CLB (1979). A study of craniofacial form. Angle Orthod.

[13] Battagel JM (1993). The aetiological factors in Class III
malocclusion. Eur J Orthod.

[14] Moss ML (1956). Malformations of skull base associated with cleft palate
deformity. Plast Reconstr Surg (1946).

[15] Dalh E (1970). Craniofacial morphology in congenital clefts of lip and
palate. Acta Odontol Scand.

[16] Hoswell BB, Gallup BV (1992). Base of the skull morphology in cleft lip and palate: A
cephalometric study from 7 to 18 years of age. J Oral Maxillofac Surg.

[17] Harris EF (1993). Size and form of base of the skull in isolated cleft lip
and palate. Cleft Palate Craniofac J.

[18] Cortés J, Granic X (2006). Characteristic craniofacial features in a group of
unilateral cleft lip and palate patients in Chile. Rev Stomatol Chir Maxillofac.

[19] Brader AC (1957). A cephalometric appraisal of morphological variations in
base of the skull and associated pharyngeal structures. Angle Orthod.

[20] Ross RB (1965). Base of the skull in children with cleft lip and
palate. Cleft Palate J.

[21] Chierici G, Harvold E, Vargervik K (1973). Morphogenetic experiments in cleft palate: mandibular
response. Cleft Palate J.

[22] Velemínská J (2000). Analysis of intracranial relations in patients with
unilateral cleft lip and palate using cluster and factor analysis. Acta Chir Plast.

[23] Jacobson A (1995). Radiographic Cephalometry.

[24] Nahler G (2009). Dictionary of Pharmaceutical Medicine.

[25] Bishara SE, Iversen WW (1974). Cephalometric comparisons on the base of the skull and
face in ndividuals with isolated clefts of the palate. Cleft Palate J.

[26] Krogman WM, Mazaheri M, Harding RL, Ishiguro K, Bariana G, Meier J (1975). A longitudinal study of craniofacial growth pattern in
children with clefts as compared to normal, birth to six years. Cleft Palate J.

[27] Sandham A, Cheng L (1988). Base of the skull and cleft lip and
palate. Angle Orthod.

[28] Trotman CA, Collett AR, McNamara JA, Cohen SR (1993). Analyses of craniofacial and dental morphology in
monozygotic twins discordant for cleft lip and unilateral cleft lip and
palate. Angle Orthod.

[29] Mestre JC, De Jesus J, Subtelny JD (1960). Unoperated oral clefts at maturation. Angle Orthod.

[30] Huang CS, Wang WI, Liou EJ, Chen YR, Chen PK, Noordhoff MS (2002). Effects of cheiloplasty on maxillary dental arch
development in infants with unilateral complete cleft lip and
palate. Cleft Palate Craniofac J.

[31] Singh GD, Rivera-Robles J, de Jesus-Vinas J (2004). Longitudinal craniofacial growth patterns in patients
with orofacial clefts: geometric morphometrics. Cleft Palate Craniofac J.

[32] Liao YF, Mars M (2005). Long-term effects of lip repair on dentofacial
morphology in patients with unilateral cleft lip and palate. Cleft Palate Craniofac J.

[33] Corbo M, Dujardin T, de Maertelaer V, Malevez C, Glineur R (2005). Dentocraniofacial morphology of 21 patients with
unilateral cleft lip and palate: a cephalometric study. Cleft Palate Craniofac J.

[34] Hagerty RF, Hill MJ (1963). Facial growth and dentition in the unoperated cleft
palate. Fissurados. J Dent Res.

[35] Blaine HL (1969). Differential analysis of palate
anomalies. J Dent Res.

[36] Capelozza L, Normando AD, Silva OG (1996). Isolated influences of lip and palate surgery on facial
growth: Comparison of operated and inoperated male adults with
UCLP. Cleft Palate Craniofac J.

[37] Silva OG, Calvano F, Assunção AG, Cavassan AO (2001). Craniofacial morphology in children with complete
unilateral cleft lip and palate: a comparison of two surgical
protocols. Angle Orthod.

[38] Tanabe Y, Taguchi Y, Noda T (2002). Relationship between base of the skull structure and
maxillofacial components in children aged 3-5 years. Eur J Orthod.

[39] Andria LM, Leite LP, Prevatte TM, King LB (2004). Correlation of the base of the skull angle and its
components with others dental/skeletal variables and treatment
time. Angle Orthod.

